# One Group's Historical Reflections on DNA Vaccine Development

**DOI:** 10.1089/hum.2018.066

**Published:** 2018-09-17

**Authors:** Ellen F. Fynan, Shan Lu, Harriet L. Robinson

**Affiliations:** ^1^ Department of Biology, Worcester State College, Worcester, Massachusetts.; ^2^ Department of Medicine, University of Massachusetts Medical School, Worcester, Massachusetts.; ^3^ GeoVax, Inc., Smyrna, Georgia.

**Keywords:** DNA vaccines, antibody, cytotoxic T cells, heterologous prime-boost

## Abstract

DNA vaccines were pioneered by several groups in the early 1990s. This article presents the reflections of one of these groups on their work with retroviral vectors in chickens that contributed to the discovery and early development of DNA vaccines. Although the findings were initially met with skepticism, the work presented here combined with that of others founded a new method of vaccination: the direct inoculation of purified DNA encoding the target antigen.

In 1992, our laboratory was one of the pioneers on the use of *in vivo* DNA-expressed proteins to elicit protective immune responses. As with many novel concepts, this “radical” method of vaccination met with skepticism and doubt. Jenner self-published his use of variolation to protect against smallpox because the Royal Society considered that they might damage the Society's reputation by publishing his findings in the Proceedings of the Royal Society.^1^ So too, the idea of using DNA as a vaccine was first considered questionable. However, convincing experimental evidence from our laboratory and others over the past 25 years has demonstrated the powerful potential of this method for immunization and contributed to the use of *in vivo* expression of DNA-encoded proteins for gene therapy, cancer immunotherapy, and monoclonal antibody production.^2–4^

The development of live vaccinia virus as an expression vector and its use as a vaccine in 1982 generated interest in the use of viral vectors for vaccination.^5,6^ Recognizing the potential of this method and possible extension to avian diseases, our group inserted the gene for avian influenza hemagglutinin, the major target for protective antibody, into a replication-competent avian retrovirus vector.^7^ Transfection of the recombinant retroviral vector into chick embryo fibroblasts resulted in production of the vector and expression of the influenza hemagglutinin insert for >2 weeks. In experiments conducted in collaboration with Rob Webster of St. Jude's Children's Research Hospital (which had the appropriate BSL3 laboratory for testing avian influenza virus infections in chickens), the retroviral vector–based vaccine completely protected chickens against a lethal influenza virus challenge.^7^ In contrast, birds within the control group succumbed to influenza. Given this, we next tested an infectious, replication-defective pseudotype of the retroviral vector for the ability to provide protection. This replication-defective pseudotype, despite inoculating <1 × 10^6^ infectious units, also achieved 100% protection, demonstrating that even low titers of a replication-defective vector could achieve protective immunity.

Retroviruses have DNA and RNA forms of their genetic information: RNA in infectious virus and DNA in infected cells. Given the ability of relatively few infectious units of the infectious, replication defective pseudotype to achieve protection and a growing body of evidence for successful *in vivo* transfection,^8,9^ we tested the ability of the DNA forms of both the replication-competent and replication-defective vectors to achieve protection. We made as much DNA as we could and asked Rob to vaccinate chickens with 300 μg of vaccine DNA or empty vector DNA (the control). Each chicken received 300 μg of DNA at weeks 0 and 4 delivered by three routes (subcutaneous, intraperitoneal, and intravenous). A lethal influenza virus challenge was administered at week 5. We learned that our first DNA experiment had worked when Rob left the message “Send more vaccine.” We had achieved 100% protection in both groups of chickens, receiving either the replication-competent or the replication-defective vectors. We immediately set out to repeat the trial, telling nobody of the result until a patent had been filed. Once we had filed, we began to present the results, but these were met with disdain and skepticism. The first question at the summer 1992 American Society of Virology meeting was “You don't think this will ever be useful, do you?” Our grants were triaged and our manuscripts returned (despite *Nature* sending the report to multiple reviewers). Fortunately, our department chair, Guido Majno, a pathologist with broad interests in the history of science and medicine and author of the bestselling book, *The Healing Hand*, recognized the potential of what we were doing and provided departmental funds to keep us going. We knew we were onto something, and we kept going.

The new technology first achieved public acceptance at the fall 1992 Cold Spring Harbor Vaccine meeting, “Modern Approaches to New Vaccines,” which was attended by a number of the early players in DNA vaccines. We presented our protective studies in chickens and mice. Margaret Liu, Jeff Ulmer, and John Donnelly of Merck showed that protective cytotoxic T cells could be elicited, David Weiner from the University of Pennsylvania described the generation of Ab responses for human immunodeficiency virus type 1 (HIV-1), and researchers from Vical presented their results on introducing DNA into muscle. The attendees clustered around the DNA posters. The field of DNA vaccines had been born! That fall, the Department of Agriculture awarded our first funding for DNA vaccines, and Shan Lu, a new postdoctoral fellow in the lab, received a Howard Hughes fellowship for studying DNA-based immunizations. It would, however, take another year and increasing sophistication in immunology on our part to “merit” National Institutes of Health funding.

Taken together, it was becoming clear that transfection could occur *in vivo* and that low numbers of cells expressing a plasmid were sufficient to stimulate an immune response. However, given the concern that an endogenous virus might render our replication-defective retroviral vectors infectious, we undertook *in vivo* antigen expression with a non-retroviral DNA vector, comprised of a mammalian expression plasmid with the gene for the influenza hemagglutinin antigen under the control of a strong eukaryotic promoter. These studies readily replicated the success achieved with the retroviral vectors.

With protection against disease shown in DNA-vaccinated chickens, we moved our studies into much more tractable mouse models. Influenza hemagglutinin expressing plasmid DNA successfully protected BALB/c mice following intramuscular and intravenous inoculations using a hypodermic needle and syringe; intranasal inoculations, using nose drops; and epidermal inoculations using a gene gun. A prototype gene gun (Accell^®^) was acquired from Agracetus (Middleton, WI) where it had been developed primarily to introduce DNA into plant cells and, later, live animals.^10–12^ In our experiments, we used the gene gun to blast gold particles coated with the plasmid DNA into the shaved abdominal skin of mice. In earlier biolistic studies, Stephen Johnston had used a gene gun to deliver human growth hormone to the outer ears of mice and realized that he had not affected mouse growth but had elicited Ab to human growth hormone.^13^ The use of the Agracetus gun (the size of a refrigerator) generated a great deal of excitement (and noise) within the department, but did not allay suspicions about our laboratory's endeavors.

Our initial experiments in mice were highly successful: 95% of the mice inoculated intramuscularly survived the lethal influenza virus challenge. Even more striking were the results of the gene gun inoculations. Mice were protected against an influenza challenge virus with 250–2,500 times less plasmid DNA than with the other routes of administration.^14^ These results—along with pioneering work by Jon Wolff of the University of Wisconsin and Phillip Felgner of Vical on intramuscular delivery of DNA;^15^ Margaret Liu, Jeff Ulmer, and John Donnelly at Merck, which had licensed the delivery of “naked DNA” to muscle from Vical;^16^ Stephen Johnston of the Southwestern Medical Center on ballistic delivery of DNA to elicit Ab;^13,16^ David Weiner of the University of Pennsylvania;^17^ Heather Davis and Bob Whalen of the Pasteur Institute;^18,19^ Hildegund Ertl of the Wistar Institute;^20^ and Britta Wahren of the Karolinska Institute^19^—gained acceptance for this new vaccination method and encouraged others to try this novel avenue of vaccine research.^21^

Given the ease of DNA vaccine construction and manufacture, early DNA vaccines had reached the clinic within 5 years of the first demonstration of *in vivo* immunogenicity (Table 1). Despite the extraordinarily rapid reduction of this new technique to clinical use, we are now 25 years out and do not have a single licensed DNA vaccine for humans. Part of this is due to DNA vaccines having been used for the development of vaccines for chronic infections, such as HIV, tuberculosis, and malaria, which are difficult targets for vaccination. A second important factor is the low level of immune responses that are elicited by DNA vaccines when used alone or without assisted delivery such as by electroporation.

**Table 1.** Timeline for DNA vaccines

1992	Demonstration of the ability to elicit antibody^a^
1993	First protective studies in animals
1994	Naming of technology, WHO^b^
1995	First prophylactic Phase I human trial^c^
1996	FDA points to consider for DNA-based vaccines^d^
1998	HIV, malaria, influenza, herpes, and hepatitis B virus vaccines in clinical trials

^a^ Demonstrated in a “gene therapy” experiment in which human growth hormone was being delivered to mice to enhance growth.

^b^ Names under consideration included genetic immunization, polynucleotide immunization, gene vaccines, and DNA vaccines.

^c^ This first prophylactic trial, a Merck plasmid expressing influenza hemagglutinin, was never published due to it not having worked and Mary Lou Clements-Mann of Johns Hopkins, its P.I., having died in the crash of Swiss Air, flight 111.

^d^ Points to consider present guidelines for the manufacture of vaccines.

WHO, World Health Organization; FDA, Food and Drug Administration; HIV, human immunodeficiency virus.

The elicitation of low-titer antibody responses was evident in our earliest experiments in chicken where large amounts of DNA (300 μg of DNA) raised essentially undetectable Ab responses. The “undetectable” Ab responses did undergo strong anamnestic expansions post challenge, sufficiently strong to protect against infections that could kill within a week of challenge. These strong anamnestic responses differed from anamnestic responses primed by a natural infection by being focused on the antigen primed by the vaccine, rather than the totality of the immunogenic proteins of an infection.^22^

This characteristic of DNA vaccines, the elicitation of low but specific responses, is the foundation for the currently popular use of DNA to prime responses that are then boosted by a live vector, peptide, or protein.^23^ In these heterologous prime-boost regimens, the DNA focuses the immune response on its vaccine insert, promotes antigen-specific B-cell development at the germinal center of lymph nodes, and activates the innate immune system to promote acquired immunity.^24,25^ Live vectors such as modified vaccinia Ankara are then used to boost the memory response for the vaccine antigens, which occurs largely to the exclusion of the antigens present in the boosting vector. Protein boosts are most effective for the epitopes that are common to the DNA-expressed antigen and the boost. In this case, the DNA-expressed antigen can serve to focus the boost on epitopes that represent the native antigen. These are readily preserved on DNA-expressed proteins but trickier to preserve on protein immunogens that need to undergo production, purification, and storage.

Heterologous prime boosts can be highly effective for eliciting high levels of CD8^+^ T cells^26,27^ and antibody.^28,29^ They are, however, cumbersome for the development of real-world vaccines because they require two different products that need to be used in the correct order. Thus, they can be expensive to manufacture and deliver. Recently, however, successful vaccination, including the generation of neutralizing Ab, has been achieved in nonhuman primates with a DNA vaccine for Zika virus using only two intramuscular bioject deliveries of the vaccine preparation.^30^ Following challenge, 95% (17/18) of the animals had no detectable viremia. Currently, this Zika virus DNA vaccine candidate is in Phase I clinical trials (ClinicalTrials.gov NCT02840487). This success reflects the high immunogenicity of the Zika glycoprotein, and sets a precedent for DNA immunizations with other highly immunogenic proteins holding good promise for success. Following electroporation, Ebola, Marburg, and Middle East respiratory syndrome (MERS) vaccines have shown promise in nonhuman primates,^31,32^ and the MERS vaccine has been advanced to clinical trials (ClinicalTrials.gov NCT02670187).

As DNA vaccines have undergone development, many advances have been made in DNA expression and delivery. Jet injectors,^33^ improved liposomes,^34,35^ and electroporation^36^ have enhanced responses through increased efficiency of DNA delivery. Work on expression cassettes has identified promoters, enhancers, and introns that optimize responses.^37^ Pathogen genes have been codon optimized for human usage to enhance expression.^38^ The elicitation of immune responses has been modulated by removing (to tolerize)^39^ or increasing (to enhance)^40^ the CpG motifs in plasmid DNA that stimulate innate responses.^41^ Extensive studies have employed genetic (DNA-encoded) adjuvants to enhance and shape immune responses.^20,42^ Gene guns, however, have not advanced from being a tool suited to the laboratory to general use so far, but there is renewed effort by Deborah Fuller from the University of Washington on this approach.

As for the authors, we are still using DNA for vaccination. Two of us are using heterologous prime-boost regimens for the development of a HIV vaccine. One of us (H.L.R.) is using the DNA as a prime for modified vaccinia Ankara boosts.^43–45^ In this case, the DNA facilitates the display of the native form of the HIV Env on virus like particles. Another (S.L.) is using DNA as a prime for gp120 protein subunit boosts as part of a polyvalent HIV vaccine strategy. The last (E.F.F.) is teaching the next generation of experimental biologists.
